# A comprehensive analysis of excess depressive disorder in women and men living with HIV in France compared to the general population

**DOI:** 10.1038/s41598-022-10263-3

**Published:** 2022-04-16

**Authors:** Victor Hémar, Mojgan Hessamfar, Didier Neau, Marc-Olivier Vareil, Nicolas Rouanes, Estibaliz Lazaro, Pierre Duffau, Charles Cazanave, Patrick Rispal, Valérie Gaborieau, Olivier Leleux, Linda Wittkop, Fabrice Bonnet, Diana Barger

**Affiliations:** 1 Team MORPH3EUS, UMR 1219, CIC-EC 1401, ISPED, Inserm, Bordeaux Population Health Research Center, University Bordeaux, Bordeaux, France; 2 Service de Médecine Interne et Maladies Infectieuses, Bordeaux University Hospital-Saint-André, Bordeaux, France; 3 Bordeaux University Hospital-COREVIH Nouvelle Aquitaine, Bordeaux, France; 4 Service des Maladies Infectieuses et Tropicales, Bordeaux University Hospital-Hôpital Pellegrin, Bordeaux, France; 5 Service de Maladies Infectieuses, Côte Basque Hospital, Bayonne, France; 6 Service de Médecine Polyvalente, Centre Hospitalier de Périgueux, Périgueux Hospital, Périgueux, France; 7 Service de Médecine Interne, Bordeaux University Hospital-Haut-Lévêque, Pessac, France; 8 UMR 5164, ImmunoConcEpT, Department of Immunology, CNRS, Bordeaux, France; 9 Service de Médecine Interne, Agen-Nérac Hospital, Agen, France; 10 Service de Maladies Infectieuses, Pau Hospital, Pau, France; 11 Pôle de Santé Publique, Bordeaux University Hopital, Bordeaux, France; 12 Team EMOS, UMR 1219, ISPED, Inserm, Bordeaux Population Health Research Center, University Bordeaux, Bordeaux, France; 13 Service de Médecine Interne et Maladies Infectieuses, Hôpital Saint-André, 1 Rue Jean Burguet, 33000 Bordeaux, France

**Keywords:** Epidemiology, Viral infection, Depression

## Abstract

We aimed to estimate the prevalence of depressive disorder in people living with HIV (PLWH) and evaluate its association with non-HIV-specific and HIV-specific factors in PLWH and in PLWH compared to the general population (GP). We used cross-sectional data from the QuAliV study, conducted within the ANRS-CO3 Aquitaine-AQUIVIH-NA cohort of PLWH in Nouvelle-Aquitaine (2018–2020), and a nationally-representative survey in the GP (EHIS-ESPS, 2014–2015), we included all participants aged ≥ 18 years old who had completed the Patient Health Questionnaire-8 (PHQ-8). Depressive disorder was defined as Patient Health Questionnaire-8 score greater or equal to 10. Its association with non-HIV-specific (demographic, socio-economic, behavioral, health status), HIV-specific factors (immuno-viral markers, antiretrovirals, level of perceived HIV-stigma), and HIV-status was assessed using Poisson regression models with robust variance in women and men separately. We included 914 PLWH (683 men/231 women). More than one in five PLWH had depressive disorder. It was strongly associated with being younger and experiencing severe pain in both sexes. Unemployment in women, being single, and lack of family ties in men were also associated with depressive disorder. More than 30% of our sample reported HIV-stigma, with a dose–response relationship between level of perceived HIV-stigma and depressive disorder. The crude prevalence of depressive disorder was 2.49 (95%CI 1.92–3.22) and 4.20 (95%CI 3.48–5.05) times higher in women and men living with HIV respectively compared to GP counterparts and 1.46 (95%CI 1.09–1.95) and 2.45 (95%CI 1.93–3.09) times higher after adjustment for non-HIV specific factors. The adjusted prevalence ratio of depressive disorder was not significantly different in HIV-stigma free women, but remained twice as high in HIV-stigma free men. The prevalence of depressive disorder compared to the GP tended to decrease with age in PLWH. Excess depressive disorder remains a major concern in PLWH. Our findings reaffirm the importance of regular screening. Tackling social inequalities and HIV-stigma should be prioritized to ensure that PLWH achieve good mental as well as physical health outcomes.

## Introduction

Human immunodeficiency virus (HIV) has gone from a fatal infection to a chronic disease thanks to improved screening and access to effective antiretroviral therapy (ART)^1,2^. However, people living with HIV (PLWH) have lower health-related quality of life (QoL) than the general population^3^. Where the UNAIDS 2020 “90–90–90” targets have been achieved, a fourth 90, “ensuring good health-related QoL in 90% of those who have achieved viral suppression” has been proposed as the ultimate goal of HIV care^4^. Depressive disorder impairs QoL in PLWH^5^. It affects ART adherence, hindering viral control and immune restoration, and is associated with cognitive impairment and all-cause mortality^6–8^. In France, over a decade ago, the prevalence of depressive disorder was estimated at 28.1% in PLWH or 2 to 5 times greater than in the general population (GP), varying by HIV risk group^9^. This observed excess of depressive disorder in PLWH has been partially attributed to non-HIV-specific factors, specifically poorer socio-economic conditions, social isolation, substance use disorders, and/or multi-morbidity^10,11^. Yet, the role of HIV infection specifically in the pathophysiology of depressive disorder remains poorly understood. Some have implicated the neurotropic viral effect on inflammation and dopaminergic transmission or neuropsychiatric effects of integrase inhibitors and efavirenz^12^. In addition to the potential direct viral and antiretroviral influence on mood, stigma or “the social process in which individuals with socially undesirable attributes or identities are seen as having lower social value than others and as a consequence face prejudice and discrimination” may also pose a threat to PLWH’s health. Individual HIV-related stigma is a multidimensional concept which has been elucidated by Turan et al. as including perceived community stigma, experienced stigma, internalized stigma and anticipated stigma^13^. The association between stigma and HIV-related stigma specifically and health outcomes is premised on the idea that those who are socially marginalized (because of their race/ethnicity, sexual orientation or HIV serostatus or a combination of the above) experience more social stress than their non-marginalized counterparts. HIV-related stigma has been hypothesized to manifest via poor access to resources, a lack of social support, maladaptive psychological and behavioural responses and physiological stress, resulting in authentic depressive disorder in PLWH^14^.

The HIV prevention, treatment and care landscape has nevertheless evolved, e.g. immediate ART initiation (fewer HIV-related complications), new more well-tolerated classes of ART, and campaigns targeting HIV-stigma^15,16^, potentially easing the burden of depressive disorder in PLWH. Thus, we aimed to (1) estimate the prevalence of depressive disorder in PLWH, (2) identify factors associated with depressive disorder in PLWH, and (3) understand which factors are associated with an excess of depressive disorder in PLWH compared to the GP.

## Methods

We conducted the QuAliV- “Depressive Disorder” study (QuAliV-DD) using data from the ANRS CO3 Aquitaine-AQUIVIH-NA cohort and the QuAliV study. Launched in 1987, the former is an open, prospective, hospital-based cohort of HIV-1 diagnosed adults (≥ 18 years old [y.o.]) in care in 15 public hospitals in south-western France. Detailed demographic, epidemiological, clinical and laboratory data are collected from participants’ medical records, standardised and entered into a locally-developed electronic Case Report Form. Diagnoses and prescribed treatments are recorded using standard national and international nomenclature. Patient-reported outcomes were introduced via the QuAliV study in 2018^17^. Cohort participants are invited to complete an electronic or identical pen-and-paper Patient-reported outcomes assessment independently at their hospital-based HIV consultation.

The EHIS-ESPS survey is conducted in a representative, randomly-selected, sample of French national health insurance beneficiaries every 2 years^18^. Data are collected through structured face-to-face or telephone interviews, resulting in a sample that reflects 95% of the population. Participants aged > 15 years old completed a self-administered questionnaire (75% participation rate) covering their demographic, socio-economic, behavioral characteristics and general health status which includes their recent (past 12 months) history of common chronic illnesses. The present study draws on the most recent available data, collected in 2014–2015.

### Study populations

We considered all cohort participants seen at a hospital-based HIV consultation between the 1/1/2018 and 12/31/2020 as our source population. We performed analyses in a sub-sample of QuAliV participants who had completed the Patient Health Questionnaire 8 (PHQ-8) prior to 1 March 2020 to avoid a “period effect” due to COVID-19 pandemic restriction^19^. In comparative analyses of PLWH versus the GP, we included participants aged ≥ 18 years old who had completed the PHQ-8 and excluded PLWH who were homeless (as they were excluded of EHIS-ESPS survey) and GP participants who were students as they were largely underrepresented among PLWH. As HIV status was not available in EHIS-ESPS survey, we assumed that participants were HIV-negative given the very low prevalence (< 1%) of HIV in France^9^.

### Outcome: depressive disorders

The PHQ-8 was used to measure depressive disorder in PLWH and the GP. It is a valid diagnostic and severity measure wherein the frequency of eight symptoms (out of nine), used to diagnose depressive disorder according to the Diagnostic and Statistical Manual of Mental Disorders-V (DSM-V), is rated, generating a score (0–24), with higher scores indicating more severe depressive symptoms. We applied a cut-off of ≥ 10 to classify clinically meaningful current depressive disorder^20^. This self-administered screening instrument has been widely used in epidemiological studies. It does not, however, distinguish between the clinical subtypes of depressive disorder as classified in the DSM-V.

### Non HIV-specific factors

We identified variables a priori based on the literature and included them in our analysis depending on their availability. We considered participants’ demographic, socio-economic, behavioral characteristics and their general health status. We considered participants’ age, place of origin and sexual orientation, highest level of education, employment status, net monthly household income/consumption unit, and partnership status. We defined a lack of family ties as having no contact with a family member in the past 6 months (other than children, parents) and a lack of social support as not having someone who could put them up for a few days. We defined alcohol misuse as an Alcohol Use Disorders Identification Test-Consumption (AUDIT-C) score of ≥ 4 in men and ≥ 3 in women^21^. Cannabis and recreational drug use in the past 12 months (none, less or more than once a month) was only considered in analyses in PLWH (not collected in the EHIS-ESPS). The question “Has physical pain made it difficult for you to do your daily activities in the last two weeks?” was used to assess pain severity. We considered multimorbidity as the number of age-associated comorbidities (none to ≥ 3 comorbidities). In analyses conducted exclusively in PLWH, we considered commonly occurring comorbidities in PLWH as per previous analyses^11,22^. These included hypertension, diabetes mellitus type 2, chronic obstructive pulmonary disease, ischemic cardiac event (angina pectoris or myocardial infarction), ischemic cerebrovascular disease as well as impaired renal function, osteoporosis, peripheral arterial disease, and non-AIDS cancers, which were not available in the EHIS-ESPS survey. In analyses comparing PLWH and the GP, we modified the definition of multimorbidity, considering only hypertension, diabetes mellitus type 2, chronic obstructive pulmonary disease, ischemic cardiac events (angina pectoris or myocardial infarction), and ischemic cerebrovascular diseases.

### HIV-specific factors

We considered participants’ history of HIV disease severity based on the 1993 Revised Centers for Disease Control Revised Classification System for HIV Infection^23^, time since HIV diagnosis, a history of hepatitis C virus co-infection (at least one positive serology/RNA) and current ART regimen defined according to the third antiretroviral agent, typically prescribed in addition to a common backbone of two nucleosidic reverse transcriptase inhibitor. We used routine measures of immunological and viral status taken within a ± 2-year interval of participation in the QuAliV study. HIV-stigma was evaluated with the question : “To what extent are you bothered by people blaming you for your HIV status?”, which is an item in the WHOQOL-HIV-BREF questionnaire, a validated instrument designed to assess multidimensional quality of life in PLWH^24^. If participants responded “not at all”, “a little”/ “a moderate amount”, or “very much”/ “an extreme amount”, we assumed that they experienced “none”, “moderate” or “severe” perceived HIV-stigma respectively given the phrasing of this question^14^.

### Statistical analysis

All analyses were conducted using R Studio (Version 1.1.463). We estimated crude and weighted prevalence of depressive disorder in PLWH to account for non-random sampling and non-response. Weights for age categories, HIV risk group, and place of origin were derived from those having at least one HIV consultation between 1/1/2018 and 12/31/2020, representing 5533 people in care in the region. We performed calibration on margins using the Icarus package in R^25^.

We stratified all analyses by sex because of expected differences in prevalence of depressive disorder, the distribution of covariates and unmeasured factors affecting men and women differentially (structural inequalities affecting power and access to resources, violence, hormonal effects). We described participants’ characteristics with frequencies and percentages for categorical variables and medians and interquartile range for continuous variables.

We performed bivariable analyses using Chi-2 tests for categorical variables and Wilcoxon tests for continuous variables and multivariable analyses using a-priori-defined block-entry Poisson regression modelling with robust variance error estimation^26^, which allowed us to investigate associations between blocks of variables and better understand the effects of confounding factors. A p-value of < 0.05 was considered statistically significant. To account for missing data, we performed Multivariate Imputation by Chained Equations, assuming that data were Missing-At-Random. We created 10 imputed datasets using logistic and linear regression models, including all variables in subsequent analysis as independent variables. All analyses were performed on imputed datasets^27^.

In the analysis conducted in PLWH (“PLWH analysis”), blocks of variables were defined in multivariable analyses as follows: demographic (Model A), plus socio-economic (Model B), plus behavioral (Model C) and plus health status (Model D). We assessed associations between HIV-specific factors and depressive disorder individually by adding each variable separately to Model D. HIV-specific factors found to be significantly associated informed comparative analyses.

In the “PLWH versus GP analysis”, we performed analyses treating the main explanatory variable (HIV status) as both a binary and categorical variable (stratified by level of HIV-stigma). Blocks were defined as follows: Model 0: unadjusted, Model 1: adjusted for age, Model 2: plus socio-economic variables, Model 3: plus partnership status; Model 4: plus family ties; Model 5: plus alcohol use, Model 6: plus age-related comorbidities, Model 7: plus pain status.

In both analyses, we handled non-linearity of continuous variables by applying cubic regression splines. We tested whether there was an interaction between age group and perceived HIV-stigma. Sensitivity analyses to assess the robustness of our findings were performed (complete case analysis [“PLWH” and “PLWH versus GP analysis”] and restricted by transmission group: “without intravenous drug use” and MSM [“PLWH versus GP analysis”]).

### Ethics approval and consent

All methods were carried out in accordance with relevant guidelines and regulations. An Institutional Review Board (Comité de Protection des Personnes Sud-Ouest et Outre Mer III) approved the ANRS CO3 Aquitaine-AQUIVIH-NA cohort study on May 27, 2016 and the QuAliV study was granted ethical approval in August 30, 2017. The National Commission on Informatics and Liberty (CNIL), the French regulatory agency charged with enforcing data privacy laws, reviewed and approved QuAliV study-specific amendments to authorisations on March 12, 2018. Informed consent was obtained from all study participants.

## Results

**Figure 1:**
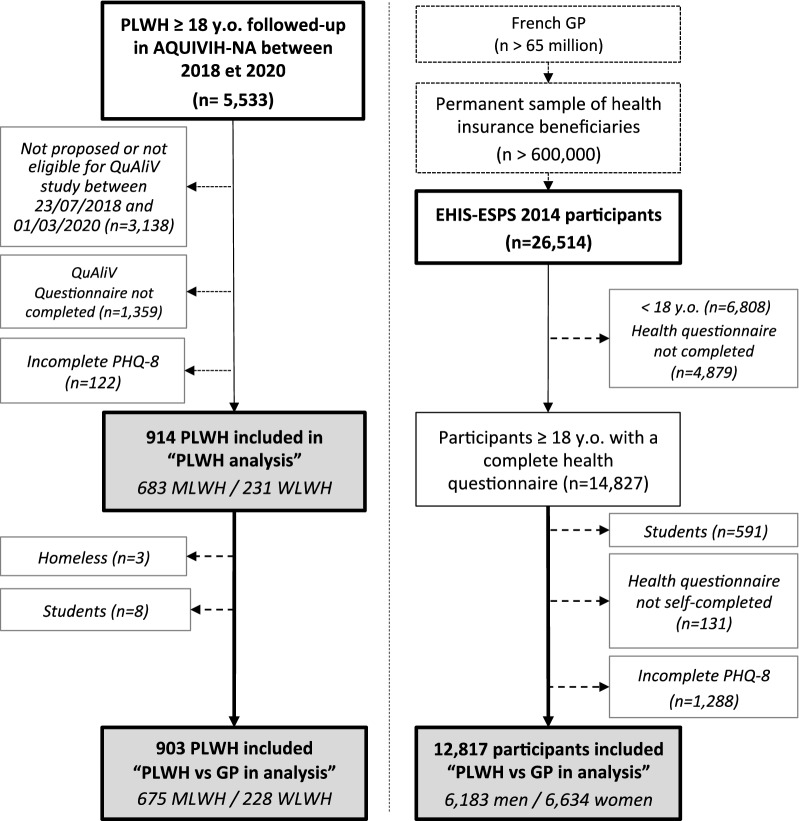
Flow chart of included patient in first and second analysis of QuAliV-DD study. *GP *general population*, HIV *human immunodeficiency virus*, PLWH *people living with HIV*, PHQ8 *Patient Health Questionnaire-8*, y.o. *years old. Microsoft PowerPoint 2011, version 14.1.0 was used to create this figure.

Among the 5,533 PLWH actively followed in ANRS C03 Aquitaine-AQUIVIH-NA cohort between 2018 and 2020, 231 women and 683 men living with HIV (W/MLWH) were considered for the “PLWH analysis” (Fig. 1). QuAliV-DD participants were slightly older, more often of French-descent and MLWH were more often MSM compared to those actively followed in the cohort (Supplementary Table 1). In WLWH, 78.4% had contracted HIV through heterosexual contact and 68.8% were of French descent, median age was 54 [IQR 48–59]. They had been diagnosed with HIV for 23 years [IQR 14–28]. In MLWH, 66.3% of whom were MSM and 92.5% of whom were of French descent, median age was 56 [IQR 48–63]. They had been diagnosed with HIV for a median of 21 years [IQR 11–28]. The most common third antiretroviral agent class was integrase inhibitors (54.9% in men and 51.1% in women) and viral suppression had been achieved by > 93% of W/MLWH. Crude depressive disorder prevalence was 22.5% in WLWH (95%CI 17.1, 27.9) and 20.8% in MLWH (95%CI 17.8, 23.8) whereas weighted prevalence was 23.4% and 21.7% respectively.

### Non HIV-specific factors

In bivariable (Table 1) and multivariable analyses (Supplementary Table 2) of non-HIV specific factors, W/MLWH with depressive disorder were younger, more frequently of foreign descent, not partnered, unemployed, had lower income and poorer family ties and social support. Unemployment was only significantly associated with depressive disorder in women (prevalence ratio: PR 1.90; 95%CI 1.10, 3.29). This association was much less pronounced after adjusting for general health status, indicating a potentially important confounder. In both sexes, higher net monthly income appeared to be protective of depressive disorder. Strong family ties and being partnered were strongly and significantly associated with lower depressive disorder prevalence in MLWH (adjusted PR (aPR) 0.65; 95%CI 0.49, 0.88 and 0.57; 95%CI 0.41, 0.79) but not in WLWH (aPR 0.84; 95%CI 0.44, 1.58 and 0.67; 95%CI 0.38, 1.19). Globally, substance use (alcohol, cannabis, and recreational drug in men) was not associated with depressive disorder (p = 0.31 in WLWH and p = 0.64 in MLWH). Experiencing severe pain but not multimorbidity was strongly associated with depressive disorder in WLWH (aPR 2.71; 95%CI 1.23, 5.99) and particularly in MLWH (aPR 5.56; 95%CI 3.67, 8.41) (Supplementary Table 2).

**Table 1:** Socio-demographic, substance use and health characteristics of PLWH according to the presence of depressive disorder (DD), in men and women (n = 914).

	Men (n = 683)				p-value^*‡*^	Women (n = 231)				p-value^*‡*^
	Without DD (n = 541)		With DD (n = 142)			Without DD (n = 179)		With DD (n = 52)		
	N (%)^*†*^	Median [IQR]	N (%)^*†*^	Median [IQR]		N (%)^*†*^	Median [IQR]	N (%)^*†*^	Median [IQR]	
Age (years)		56 [49–63]		55 [45–60]	0.02		54 [48–60]		53 [44–58]	0.23
**Place of origin**					0.05					0.20
France	506 (93.5)		126 (88.7)			127 (70.9)		32 (61.5)		
Foreign	35 (6.5)		16 (11.3)			52 (29.1)		20 (38.5)		
**Highest level of education**					0.45					0.31
Primary	56 (10.5)		20 (14.3)			31 (17.4)		11 (21.6)		
Secondary	275 (51.4)		69 (49.3)			93 (52.2)		30 (58.8)		
University	204 (38.1)		51 (36.4)			54 (30.4)		10 (19.6)		
Missing data^†^	6 (1.1)		2 (1.4)			1 (0.6)		1 (1.9)		
**Employment status**					< 0.01					0.08
Employed	278 (52.2)		63 (45.7)			94 (53.1)		21 (41.2)		
Unemployed	86 (16.1)		42 (30.4)			50 (28.2)		23 (45.1)		
Retired	169 (31.7)		33 (23.9)			33 (18.6)		7 (13.7)		
Missing data^†^	8 (1.5)		4 (2.8)			2 (1.1)		1 (1.9)		
**Net monthly household income (€)**^*§*^					< 0.01					0.01
< 900	123 (24.9)		52 (40.3)			64 (42.4)		16 (35.6)		
900–1499	128 (25.9)		37 (28.7)			45 (29.8)		25 (55.6)		
1500–2000	114 (23.1)		23 (17.8)			31 (20.5)		3 (6.7)		
2001–3000	99 (20.0)		13 (10.1)			10 (6.6)		1 (2.2)		
3001–4000	21 (4.3)		4 (3.1)			1 (0.7)		0 (0)		
> 4000	9 (1.8)		0 (0)			0 (0)		0 (0)		
Missing data^†^	47 (8.7)		13 (9.2)			28 (15.6)		7 (13.5)		
**Partnered**	274 (53.4)		44 (33.3)		< 0.01	77 (44.3)		12 (24)		0.01
Missing data^†^	28 (5.2)		10 (7)			5 (2.8)		2 (3.8)		
**Lack of family ties**	83 (15.5)		49 (35.3)		< 0.01	21 (12.7)		12 (23.1)		0.07
Missing data^†^	7 (1.3)		3 (2.1)			13 (7.3)		0		
**Lack of social support**	129 (23.8)		54 (38.8)		< 0.01	57 (31.8)		23 (45.1)		0.15
Missing data^†^	25 (4.6)		3 (2.1)			11 (6.1)		1 (1.9)		
**Alcohol use**					0.05					0.06
None	63 (12.3)		24 (17.6)			37 (23.4)		19 (39.6)		
Without misuse	221 (43.1)		44 (32.4)			55 (34.8)		16 (33.3)		
With misuse	229 (44.6)		68 (50.0)			66 (41.8)		13 (27.1)		
Missing data^†^	28 (5.2)		6 (4.2)			21 (11.7)		4 (7.7)		
**Cannabis use**					0.04					0.22
None	427 (79.4)		99 (70.2)			154 (88.0)		39 (79.6)		
< 1 per month	33 (6.1)		16 (11.3)			7 (4.0)		2 (4.1)		
≥ 1 per month	78 (14.5)		26 (18.4)			14 (8.0)		8 (16.3)		
Missing data^†^	3 (0.6)		1 (0.7)			4 (2.2)		3 (5.8)		
**Recreational drug use**					0.04					1
None	460 (86.3)		110 (78.0)			168 (98.2)		47 (97.9)		
Only poppers	15 (2.8)		6 (4.3)			0 (0)		0 (0)		
< 1 per month	21 (3.9)		13 (9.2)			0 (0)		0 (0)		
≥ 1 per month	37 (6.9)		12 (8.5)			3 (1.8)		1 (2.1)		
Missing data^†^	8 (1.5)		1 (0.7)			8 (4.5)		4 (7.7)		
**Age-associated comorbidities**^*¶*^					0.89					0.05
0	112 (22.3)		31 (23.8)			45 (26.9)		13 (26.0)		
1	172 (34.3)		41 (31.5)			81 (48.5)		16 (32.0)		
2	121 (24.1)		30 (23.1)			29 (17.4)		12 (24.0)		
≥ 3	97 (19.3)		28 (21.5)			12 (7.2)		9 (18.0)		
Missing data^†^	39 (7.2)		12 (8.5)			12 (6.7)		2 (3.8)		
**Pain status**					< 0.01					< 0.01
None	287 (53.3)		30 (21.3)			72 (40.7)		11 (22.0)		
Moderate	219 (40.7)		69 (48.9)			92 (52.0)		26 (52.0)		
Severe	32 (5.9)		42 (29.8)			13 (7.3)		13 (26.0)		
Missing data^†^	3 (0.6)		1 (0.7)			2 (1.1)		2 (3.8)		

*DD* depressive disorder, *IQR* interquartile range.

^†^Percentages excluding missing data if present, except for percentages of "Missing data".

^‡^p-value of Chi2 or Fisher test for categorical variables and Wilcoxon for continuous variables.

^§^Net monthly household income per consumption unit CU (adult = 1CU; child > 16 = 0.5CU; child < 16 = 0.3CU).

^¶^Age-associated comorbidities: hypertension, diabetes mellitus type 2, chronic obstructive pulmonary disease, impaired renal function, ischemic cardiac event, ischemic cerebrovascular disease, peripheral arterial disease, osteoporosis, and non-AIDS cancers.

### HIV-specific factors

In bivariable (Supplementary Table 3) and multivariable analyses of HIV-specific factors and depressive disorder (Table 2), neither markers of immunological or viral status nor third agent antiretroviral classes were found to be associated with depressive disorder. Perceived HIV-stigma was the only HIV-specific factor associated with depressive disorder and a strong dose–response relationship between the level of perceived HIV-stigma and depressive disorder was observed, with said effect being more pronounced in WLWH (aPR 2.69; 95%CI 1.52, 4.76 for those with severe HIV-stigma) compared to MLWH (aPR 1.71; 95%CI 1.15, 2.53). No significant interaction was observed between perceived HIV-stigma and age (p = 0.32 in women, p = 0.25 in men). Similar results were obtained in complete case analyses.

**Table 2:** Associations between HIV-specific factors and depressive disorders (DD) in multivariable analysis, after multiple imputation of missing data, in men and women (n = 914).

	Men (n = 683)		Women (n = 231)	
	PR of DD (CI95%)^†^	p-value^†^	PR of DD (CI95%)^†^	p-value^†^
**AIDS**	1.10 (0.79–1.54)	0.57	0.78 (0.40–1.52)	0.46
**Time since HIV diagnosis**^**‡**^ **(per 3 years)**	1.02 (0.97–1.08)	0.39	0.99 (0.89–1.11)	0.90
**Nadir CD4 count (per 100cells/µL)**	1.00 (0.93–1.07)	0.93	1.01 (0.90–1.14)	0.85
< 200 vs > 200 cells/µL	1.26 (0.94–1.68)	0.07	0.99 (0.62–1.58)	0.96
**Last CD4 count (per 100cells/µL)**	1.01 (0.95–1.07)	0.79	1.05 (0.98–1.13)	0.15
< 500 vs > 500 cells/µL	1.18 (0.84–1.65)	0.34	0.61 (0.33–1.14)	0.12
**Last CD4/CD8 ratio (per 0.1 increase)**	0.99 (0.97–1.02)	0.52	0.99 (0.96–1.03)	0.64
< 1 vs > 1	1.05 (0.79–1.41)	0.73	1.05 (0.65–1.68)	0.85
**Third antiretroviral agent (ref: NNRTI)**		0.95		0.58
INI	0.93 (0.66–1.32)		0.68 (0.37–1.26)	
Others	0.95 (0.62–1.47)		0.69 (0.33–1.41)	
History of hepatitis C^*§*^	0.90 (0.57–1.43)		1.60 (0.88–2.92)	0.12
**Perceived HIV-stigma (ref: none)**		**0.02**		**0.03**
Moderate	**1.50 (1.06–2.13)**		1.74 (0.88–342)	
Severe	**1.71 (1.15–2.53)**		**2.69 (1.52–4.76)**	

*DD* depressive disorder,* INI* integrase inhibitors, *MSM* men who have sex with men, *NNRTI* non nucleosidic reverse transcriptase inhibitor,* PR* prevalence ratio (Poisson regression with robust variance).

^†^All PR are adjusted for age, place of origin, highest level of education, net monthly household income, employment status, partnership status, family ties, social support, alcohol and cannabis use, age-associated comorbidities and pain status. In men, analyses were also adjusted for MSM status and recreational drug use. Bold results are statistically significant for p-value of likelihood ratio test < 0.05.

^‡^Plus adjustment for AIDS.

^§^Plus adjustement for HIV transmission via Intravenous drug use.

### Factors associated with excess depressive disorder compared to GP

**Figure 2:**
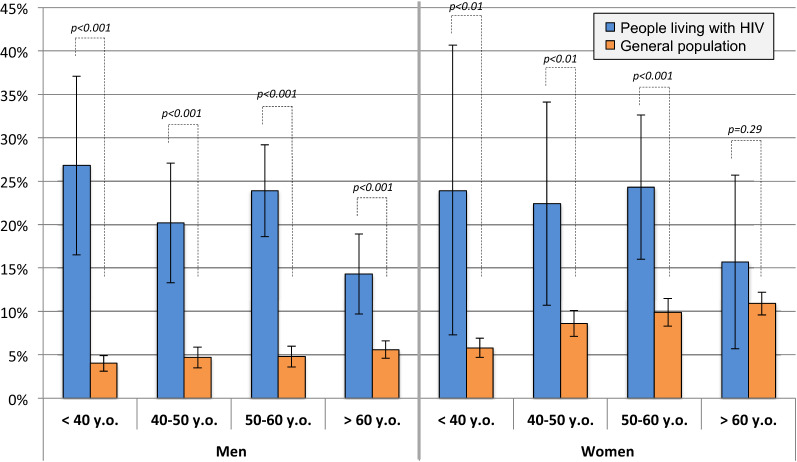
Prevalence of depressive disorder according to PHQ8 in PLWH (QuAliV-DD 2018–2020 study, n = 903), and general population (EHIS-ESPS 2014–2015 survey, n = 12,817), by age and sex. *HIV *human immunodeficiency virus*, PLWH *people living with HIV,* PHQ-8 *Patient Health Questionnaire-8*, y.o. *years old*.* p-value of Chi2 or Fisher test. Microsoft PowerPoint and Excel 2011, version 14.1.0 were used to create this figure.

903 PLWH and 12,817 EHIS-ESPS survey participants were included in “PLWH versus GP analysis” (Fig. 1). Compared to the GP, MLWH had higher levels of education but were more frequently unemployed and had lower income. WLWH reported much lower income compared to their GP counterparts. PLWH were less likely to be partnered, had weaker family ties, more comorbidities, and were more likely to report pain (Supplementary Table 4). Depressive disorder prevalence was lower in men (4.8%, 95%CI 4.3, 5.3) compared to women (8.8%, 95%CI 8.1,9.5) and increased with age in the GP whereas in PLWH, it was similar in men (20.8%, 95%CI 17.8, 23.8) and women (22.5%, 95%CI 17.1, 27.9) and decreased with age (Fig. 2).

**Figure 3:**
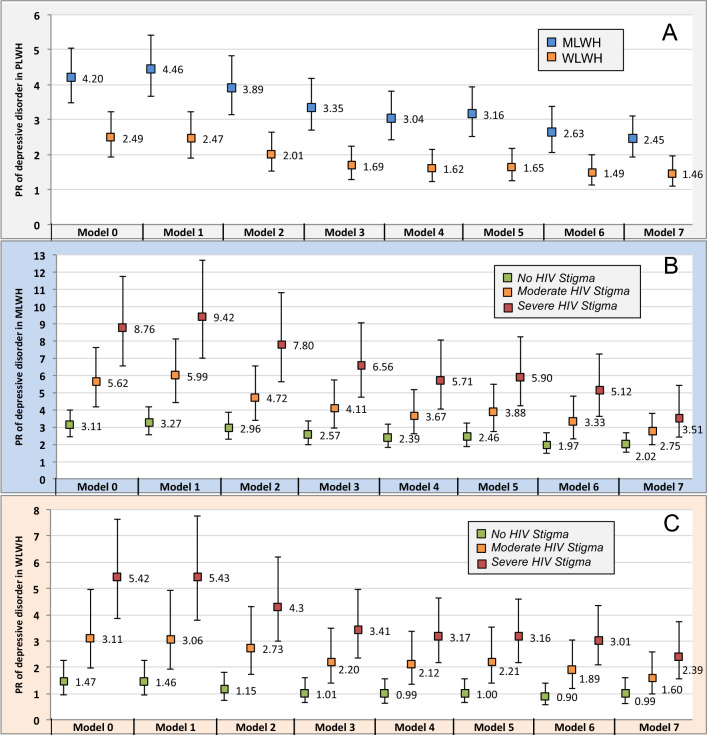
Prevalence ratio (and 95%CI) of depressive disorder in PLWH (QuAliV-DD study, n = 903), compared to general population (EHIS-ESPS survey, n = 12,817), by sex: overall **(A)** and in terms of perceived HIV-stigma in MLWH **(B**) and WLWH **(C)**, according to a-priori-defined block-entry Poisson regression with robust variance models. Model 0: unadjusted model; Model 1: adjusted for age; Model 2: Model 1 + level of education, employment status, monthly income; Model 3: Model 2 + partnership status; Model 4: Model 3 + family ties; Model 5: Model 4 + alcohol use; Model 6: Model 5 + age-associated comorbidities; Model 7: Model 6 + pain status. *95%CI *95% confidence interval, *HIV *human immunodeficiency virus*, PR *prevalence ratio*, M/WLWH *men/women living with HIV. Microsoft PowerPoint and Excel 2011, version 14.1.0 were used to create this figure.

In analyses where HIV status was defined as a binary variable, the prevalence of depressive disorder, adjusted for age (Model 1), was 2.49 (95%CI 1.92, 3.22) in WLWH and 4.20 (95%CI 3.48, 5.05) times higher in MLWH compared to the GP. The prevalence of depressive disorder in W/MLWH compared to the GP were partially confounded by socio-economic characteristics and health status (Fig. 3A, Supplementary Table 5). The PR of depressive disorder compared to the GP tended to decrease with age in WLWH (2.56 at 40 years old, 1.53 at 50 years old, 1.17 at 60 years old, p = 0.01) and slightly in MLWH (2.78 at 40 years old, 2.48 at 50 years old, 2.41 at 60 years old, p = 0.01). In analyses where PLWH were stratified according to level of perceived HIV-stigma, the prevalence of depressive disorder in PLWH who did not perceive HIV-stigma remained more than two times higher in MLWH (Fig. 3B, Model 4: aPR 2.39; 95%CI 1.82,3.15) but was no higher in WLWH than their GP counterparts (Fig. 3C, Model 4: aPR 0.99; 95%CI 0.63,1.54), (Supplementary Table 5). Results of sensitivity analyses were similar.

## Discussion

While the prognosis of PLWH has continued to improve over the last decade, 1 in 5 PLWH continue to be affected by depressive disorder in our setting. Furthermore, the crude prevalence of depressive disorder was three times higher in WLWH and four times higher in MLWH compared to their GP counterparts. The prevalence of depressive disorder remained approximately 1.5 to 2.5 times higher in women and men living with HIV respectively compared to the GP after adjustment for non-HIV-specific confounders. We also observed a dose–response relationship between the level of perceived HIV-stigma and depressive disorder in both sexes. Yet, the prevalence of depressive disorder in WLWH who did not report experiencing perceived HIV-stigma was not higher than that observed in the GP. On the contrary in MLWH, excess depressive disorder persisted even in the absence of perceived HIV-stigma in fully adjusted models, consistent with past studies in France and the United States^9,28^. These differences by sex are in part due to the higher depressive disorder prevalence in women in the GP. Another explanation requiring further investigation could be a better resilience and coping in WLWH. MLWH may be more likely than WLWH to have a history of depressive disorder, which precedes their HIV-infection. While, we were not able to investigate participants’ history of depressive disorder prior to HIV infection, Rasmussen et al., in Denmark, found antidepressant use to be 1.2 times higher in MLWH but similar in WLWH compared to those in the GP 2 years prior to HIV diagnosis^29^. Moreover, depressive disorder has been associated with the risk-taking behaviors (condom-less sex, group sex and/or Chemsex) which continue to drive new HIV infections in France^30^. Addressing the mental health needs of those at risk of contracting HIV should therefore remain a priority, especially as we promote Pre-Exposure Prophylaxis.

In 2011, in a large nationally-representative survey in France (ANRS-VESPA2), depressive disorder prevalence was estimated at 28.1%. However, it is difficult to evaluate trends between 2011 and 2018–2020 because of differences in study design, i.e. the measure of depressive disorder (CIDI-SF face-to-face by interviewer vs self-reported PHQ-8 in QuAliV-DD) and the timeframe (preceding 12-month vs 14 days)^9^. Yet, we found that the observed excess of depressive disorder in PLWH compared to the GP in 2018–2020 was, unfortunately, similar to estimates in France, the United States (2009–2010) and the Netherlands (2010–2012) a decade ago^9,31,32^. Although our non-random sample appears to be representative of those in care in the south-western France, depressive disorder prevalence may still be underestimated due to the self-reported nature of the PHQ-8, resulting in the exclusion of those with severe neuropsychiatric disorders. Furthermore, depressive disorder was reported in PLWH in 2018–2020 whereas the most recent available data in the GP was reported in 2014–2015. As trends in depressive disorder prevalence in the GP appear to be increasing, estimates of its excess presented here within may be slightly overestimated^19^. Conversely, the assumption that all EHIS-ESPS participants were living without HIV (given the very low prevalence of HIV in French GP (0.3% in 2020) may have led to a minor underestimation^33^.

Non-HIV-specific factors appear to be associated with excess depressive disorder in PLWH compared to the GP. Poor socio-economic conditions, lack of family ties, not being partnered and pain are more common in PLWH and are strongly associated with depressive disorder. With the exception of frequent cannabis use in WLWH, substance use was not associated with depressive disorder contrary to a recent British study^34^.

Depressive disorder decreased with age and the absence of an association with multimorbidity in MLWH has also been previously suggested and is encouraging as PLWH age^32,35^. Yet, the inclusion of asymptomatic as well as symptomatic comorbidities in our definition of multimorbidity may explain the lack of an association with depressive disorder. However, providers should be attentive to reported pain in PLWH given its strong association with poor mental health and likely QoL.

Markers of immunological or viral status and ART regimen were not associated with depressive disorder, contrary to earlier studies^36^. Improvement in HIV screening and care, resulting in fewer HIV-related complications, and better tolerated ART are likely to explain these results. The only HIV-specific factor found to be strongly associated with depressive disorder was moderate to severe perceived HIV-stigma, affecting more than 30% of our sample. Our HIV-stigma measure focused on two dimensions of individual HIV-stigma, enacted or perceived in the community and did not take into account either anticipated or internalized stigma that may also contribute to the development of depressive disorder. Although, our measure of perceived HIV-stigma was relatively simple compared to more comprehensive measures of HIV-stigma, the strength, consistency and gradient of the reported associations should still give providers and policymakers pause^13^. While a bi-directional relationship between the studied factors and depressive disorder is possible, especially regarding HIV-stigma, we have chosen to consider depressive disorder as the result of several determinants rather than the other way around. However, our ability to draw causal inferences is limited by the study’s design.

Under-diagnosis of depressive disorder in PLWH remains common (only 42.3% of MLWH and 38.5% WLWH with current depressive disorder had a recorded history of depression in our study) in spite of recommendations for systematic screening in the European AIDS Clinical Society guidelines^37^. These findings speak to the potential contribution of Patient-reported outcomes in the systematic screening of depressive disorder and associated factors (pain, stigma…). In two American clinics, Lawrence et al. have provided evidence of the feasibility of using routine, self-administered Patient-reported outcomes assessments in an HIV primary care setting and offered strategies for triaging those with suicidal ideations at the point-of-care^38^. Adequate investments in infrastructure and human resources to support such innovations are required.

Although France has demonstrated good linkage and retention in HIV care^39^, PLWH represent an economically and socially vulnerable population and these policies appear to have done little to address disparities in depressive disorder prevalence. In line with the latest UNAIDS Strategy which emphasises more integrated, person-centered care, underlying social inequalities must not be forgotten as we envisage the future of HIV care for PLWH.

Easing the burden of perceived HIV-stigma is a formidable challenge due to the complexity of underlying mechanisms^14^. Both structural and individual level interventions are required, such as campaigns like “Undetectable = Untransmittable” and specific messaging targeting young people and health workers, and in individuals, addressing perceived, experienced, anticipated and internalised stigma as described by Turan et al.^14,40^.

## Conclusions

Disparities in depressive disorder in PLWH compared to the GP persist in spite of improvements in the care and management of PLWH. Our findings should encourage regular screening of depressive disorder and reaffirm the importance of addressing unfavourable social and economic conditions and specifically HIV-stigma to ensure that PLWH achieve good mental as well as physical health outcomes.

## Supplementary Information

Supplementary Tables.

## Data Availability

The data that support the findings of this study are available from Diana Barger but restrictions apply to the availability of these data, which were used under license for the current study, and so are not publicly available. Data are however available from the authors upon reasonable request and with permission of Diana Barger.

## Abbreviations

AIDS: Acquired immunodeficiency syndrome
ART: Antiretroviral therapy
DSM-V: Diagnostic and Statistical Manual of Mental Disorders-V
GP: General population
HIV: Human immunodeficiency virus
IQR: Interquartile range
MSM: Men who have sex with men
MLWH: Men living with HIV
PLWH: People living with HIV
(a)PR: (Adjusted) prevalence ratio
WLWH: Women living with HIV
y.o.: Years old
