# Supplementary material for: A comprehensive analysis of excess depressive disorder in women and men living with HIV in France compared to the general population
Source: Sci Rep. 2022 Apr 16;12:6364. doi: 10.1038/s41598-022-10263-3 (PMC9013369; doi:10.1038/s41598-022-10263-3)
Supplement: Supplementary file 1 — Supplementary Tables. [file 41598_2022_10263_MOESM1_ESM.pdf]

Supplementary material

Page 2: Supplementary Table 1. Characteristics of PLWH included in “PLWH analysis” of our study (n=914) and of PLWH followed up in ANRS C03 Aquitaine-AQUIVIH-NA cohort 2018-2020 (n=5,533), in men and women.

Page 4: Supplementary Table 2. Associations between demographic, socio-economic, substance use, health status and depressive disorder (DD) in multivariable analysis, after multiple imputation of missing data, in men and women (n=914).

Page 5: Supplementary Table 3. HIV-specific factors according to the presence of depressive disorder (DD) in men and women (n=914).

Page 6: Supplementary Table 4. Socio-demographic, substance use and health characteristics of PLWH (n=903) and general population from EHIS-ESPS survey (n=12,817), in men and women included in “PLWH vs GP analysis”.

Page 8: Supplementary Table 5. Prevalence ratio of depressive disorder (DD) in PLWH (QuAliV-DD study, n=903) compared to general population (EHIS-ESPS survey, n=12,817) in men and women, pooled and by perceived HIV-stigma severity, according to a-priori-defined block-entry Poisson regression with robust variance models.

Supplementary Table 1. Characteristics of PLWH included in "PLWH analysis" of our study (n=914) and of PLWH followed up in ANRS C03 Aquitaine-AQUIVIH-NA cohort 2018-2020 (n=5,533), in men and women.

	Men				Women			
	Study population (n=683)		AQUIVIH-NA cohort (n=3,981)		Study population (n=231)		AQUIVIH-NA cohort (n=1,552)	
	N (%)[1]	Median [IQR]	N (%)[1]	Median [IQR]	N (%)[1]	Median [IQR]	N (%)[1]	Median [IQR]
**Age (years)**		56 [48-63]		55 [47-61]		54 [48-59]		52 [44-59]
<40	*78 (11.4)*		*531 (13.3)*		*26 (11.3)*		*267 (17.2)*	
40-50	*129 (18.9)*		*780 (19.6)*		*50 (21.6)*		*387 (24.9)*	
50-60	*252 (36.9)*		*1 476 (37.1)*		*104 (45.0)*		*572 (36.9)*	
>60	*224 (32.8)*		*1 194 (30.0)*		*51 (22.1)*		*326 (21.0)*	
**Place of origin**								
France	632 (92.5)		3 479 (87.4)		159 (68.8)		945 (60.9)	
Sub-Saharan Africa	17 (2.5)		182 (4.6)		56 (24.2)		466 (30.0)	
Europe	15 (2.2)		141 (3.5)		5 (2.2)		62 (4.0)	
Other countries[$]	19 (2.8)		179 (4.5)		11 (4.8)		79 (5.1)	
**HIV transmission category**								
MSM	453 (66.3)		2 372 (59.6)		/		/	
Heterosexual	125 (18.3)		910 (22.9)		181 (78.4)		1 212 (78.1)	
Intravenous Drug Use	62 (9.1)		410 (10.3)		31 (13.4)		187 (12.0)	
Other	43 (6.3)		289 (7.3)		19 (8.2)		153 (9.9)	
**AIDS stage**	133 (19.5)		804 (20.2)		38 (16.5)		283 (18.2)	
**Time since HIV diagnosis (years)**		21 [11-28]		19 [10-27]		23 [14-28]		21 [12-28]
**Nadir CD4 count (cells/µL)**		258 [136-409]		266 [137-414]		241 [118-349]		245 [128-387]
Missing data[1]	*1 (0.1)*		*13 (0.3)*		*/*		*7 (0.4)*	
**Last HIV viral load < 50 copies/mL**	637 (93.3)		3 653 (92.2)		221 (95.7)		1 414 (92.1)	
Missing data[1]	*/*		*20 (0.5)*		*/*		*16 (1)*	
**Last CD4 count (cells/µL)**		702 [526-895]		698 [503-911]		729 [544-997]		732 [517-979]
Missing data[1]	*17 (2.4)*		*91 (2.3)*		*7 (3.0)*		*36 (2.3)*	
**Last CD4/CD8 ratio**		0.95 [0.66-1.30]		0.89 [0.60-1.26]		1.29 [0.81-1.83]		1.10 [0.71-1.57]
Missing data[1]	*31 (4.5)*		*159 (4.0)*		*7 (3.0)*		*54 (3.5)*	
**Third antiretroviral agent**								
INI	375 (54.9)		2 272 (57.1)		118 (51.1)		759 (48.9)	
NNRTI	161 (23.6)		954 (24.0)		58 (25.1)		404 (26.0)	
Others	147 (21.5)		755 (19.0)		55 (23.8)		389 (25.1)	
**Age-associated comorbidities**								
0	143 (22.6)		916 (25.3)		58 (26.7)		484 (34.0)	
1	213 (33.7)		1216 (33.6)		97 (44.7)		504 (35.4)	
2	151 (23.9)		781 (21.6)		41 (18.9)		269 (18.9)	
≥3	125 (19.8)		707 (19.5)		21 (9.7)		168 (11.8)	
Missing data[1]	*51 (7.5)*		*361 (9.1)*		*14 (6.1)*		*127 (8.2)*	
**History of hepatitis C**	116 (17.5)		734 (19.1)		52 (22.5)		318 (21.6)	
Missing data[1]	*22 (3.2)*		*147 (3.7)*		*9 (3.9)*		*80 (5.2)*	
**History of depression[2]**	210 (30.7)		1 216 (30.5)		89 (38.5)		608 (39.2)	

**Current antidepressant treatment**[3]	44 (6.4)	299 (7.5)	28 (12.1)	171 (11.0)
**Current depressive disorder**	**142 (20.8)**	/	**52 (22.5)**	/

DD= depressive disorder; INI= integrase inhibitors; IQR=interquartile range; MSM= men who have sex with men; NNRTI= non nucleosidic reverse transcriptase inhibitor

[1] Percentages excluding missing data if present, except for percentages of "Missing data"

[2] According to AQUIVIH-NA cohort information

[3] Antidepressant treatment when participation to QuAliV study was proposed, or at last visit in AQUIVIH-NA cohort.

[$] Other countries: North Africa, Asia, America, Oceania.

** Age-associated comorbidities among: hypertension, diabetes mellitus type 2, chronic obstructive pulmonary disease, impaired renal function, ischemic cardiac event, ischemic cerebrovascular disease, peripheral arterial disease, osteoporosis, and non-AIDS cancers.*

Supplementary Table 2. **Associations between demographic, socio-economic, substance use, health status and depressive disorder (DD) in multivariable analysis, after multiple imputation of missing data, in men and women livng with HIV (n=914).**

	Men (n=683)				Women (n=231)			
	Model A (Demographic)	Model B (Socio-economic)	Model C (Substance use)	Model D (Health)	Model A (Demographic)	Model B (Socio-economic)	Model C (Substance use)	Model D (Health Status)
	PR of DD (95%CI)	PR of DD (95%CI)	PR of DD (95%CI)	PR of DD (95%CI)	PR of DD (95%CI)	PR of DD (95%CI)	PR of DD (95%CI)	PR of DD (95%CI)
**Age (per 10 years)**	**0.85 (0.76-0.96)**	0.87 (0.74-1.02)	0.89 (0.76-1.05)	0.87 (0.73-1.04)	0.86 (0.68-1.09)	**0.73 (0.54-1.00)**	**0.71 (0.52-0.97)**	**0.63 (0.46-0.87)**
**Born in France vs Foreign-born**	0.71 (0.44-1.12)	0.93 (0.60-1.45)	0.91 (0.58-1.44)	0.94 (0.59-1.50)	0.81 (0.48-1.35)	0.92 (0.51-1.63)	0.82 (0.45-1.49)	0.75 (0.42-1.32)
**MSM vs non-MSM**	0.89 (0.64-1.24)	1.02 (0.73-1.42)	0.99 (0.70-1.40)	1.02 (0.73-1.42)	/	/	/	/
**Level of education (ref: Secondary)**								
Primary		1.02 (0.64-1.65)	1.10 (0.67-1.81)	1.20 (0.76-1.89)		1.24 (0.68-2.25)	1.19 (0.64-2.22)	1.13 (0.59-2.17)
University		1.16 (0.84-1.60)	1.17 (0.85-1.62)	1.13 (0.83-1.54)		0.94 (0.49-1.82)	0.98 (0.52-1.86)	1.28 (0.66-2.47)
**Employment status (ref: Employed)**								
Unemployed		1.16 (0.76-1.76)	1.16 (0.75-1.78)	0.88 (0.58-1.33)		**1.90 (1.10-3.29)**	**1.73 (1.01-5.16)**	1.46 (0.88-2.40)
Retired		0.98 (0.60-1.61)	0.92 (0.56-1.54)	0.80 (0.48-1.34)		1.42 (0.57-3.53)	1.37 (0.56-3.32)	1.42 (0.59-3.46)
**Net monthly household income (€)** [1]								
<900		1.17 (0.81-1.71)	1.18 (0.80-1.74)	1.00 (0.69-1.45)		**0.40 (0.23-0.72)**	**0.40 (0.22-0.73)**	**0.49 (0.25-0.93)**
900-1499 (ref)		*1 (réf.)*	*1 (réf.)*	*1 (réf.)*		*1 (réf.)*	*1 (réf.)*	*1 (réf.)*
1500-2000 (> 1500 for women)		0.78 (0.48-1.25)	0.78 (0.48-1.26)	0.86 (0.54-1.34)		**0.27 (0.10-0.74)**	**0.29 (0.11-0.79)**	**0.30 (0.11-0.82)**
>2000		**0.58 (0.34-0.99)**	0.59 (0.35-1.01)	0.65 (0.37-1.13)		/	/	/
**Partnered (ref. Single)**		**0.58 (0.42-0.82)**	**0.57 (0.40-0.80)**	**0.57 (0.41-0.79)**		0.60 (0.32-1.14)	0.61 (0.33-1.12)	0.67(0.38-1.19)
**Family ties during last 6 months**		**0.57 (0.41-0.79)**	**0.59 (0.43-0.81)**	**0.65 (0.49-0.88)**		0.79 (0.44-1.43)	0.85 (0.46-1.54)	0.84 (0.44-1.58)
**Lack of social support**		1.24 (0.90-1.70)	1.22 (0.89-1.68)	1.10 (0.82-1.47)		1.34 (0.83-2.17)	1.35 (0.86-2.14)	1.47 (0.91-2.38)
**Alcohol use (ref: Without misuse)**								
With misuse			1.28 (0.90-1.83)	1.32 (0.96-1.81)			0.66 (0.34-1.26)	0.63 (0.33-1.20)
None			1.42 (0.91-2.23)	1.26 (0.80-1.97)			1.11 (0.3-2.88)	1.09 (0.60-1.98)
**Cannabis use (ref: None)**								
<1 per month			1.08 (0.67-1.73)	1.01 (0.63-1.60)			1.26 (0.42-3.79)	1.19 (0.36-3.95)
≥1 per month			0.81 (0.49-1.32)	0.84 (0.53-1.33)			**2.11 (1.18-3.78)**	1.88 (1.05-3.36)
**Recreational drug use (ref: None)** [3]								
Only poppers			1.33 (0.61-2.90)	1.72 (0.74-4.00)			/	/
<1 per month			1.38 (0.84-2.25)	1.53 (0.95-2.47)			/	/
≥1 per month			0.90 (0.52-1.57)	1.07 (0.61-1.87)			/	/
**Age-associated comorbidities* (ref: 0)**								
1				1.16 (0.78-1.73)				0.63 (0.34-119)
2				1.15 (0.72-1.83)				1.30 (0.63-2.67)
≥3				0.97 (0.60-1.58)				1.43 (0.62-3.30)
**Pain status (ref: None)**								
Moderate				**2.34 (1.59-3.43)**				1.81 (0.91-3.61)
Severe				**5.56 (3.67-8.41)**				**2.71 (1.23-5.99)**
p-value [2]	0.06	<0.001	0.64	<0.001	0.31	0.02	0.31	0.11

DD= depressive disorder; MSM= men who have sex with men; PR= Prevalence ratio (Poisson regression with robust variance)

[1] Net monthly household income per consumption unit CU (adult=1CU; child>16=0.5CU ; child<16= 0.3CU)

[2] p-value of Likelihood ratio test between null model and model A and then between successive nested models

[3] Recreational drug use was not included in women as only 4/231 reported any recreational drug use

** Age-associated comorbidities among: hypertension, diabetes mellitus type 2, chronic obstructive pulmonary disease, impaired renal function, ischemic cardiac event, ischemic cerebrovascular disease, peripheral arterial disease, osteoporosis, and non-AIDS cancers.*

Supplementary Table 3. HIV-specific factors according to the presence of depressive disorder (DD) in men and women (n=914).

	Men (n=683)					Women (n=231)				
	Without DD (n=541)		With DD (n=142)		p-value[2]	Without DD (n=179)		With DD (n=52)		p-value[2]
	N (%)[1]	Median [IQR]	N (%)[1]	Median [IQR]		N (%)[1]	Median [IQR]	N (%)[1]	Median [IQR]	
**Transmission category**					0.01					0.38
MSM	363 (67.1)		90 (63.4)			/		/		
Heterosexual	103 (19.0)		22 (15.5)			143 (79.9)		38 (73.1)		
Intravenous Drug Use	39 (7.2)		23 (16.2)			21 (11.7)		10 (19.2)		
Other	36 (6.7)		7 (4.9)			15 (8.4)		4 (7.7)		
**AIDS**	100 (18.5)		33 (23.2)		0.44	30 (16.8)		8 (15.4)		0.81
**Time since HIV diagnosis (years)**		20 [11-28]		21 [11-29]	0.66		24 [14-28]		21 [13-28]	0.52
**Nadir CD4 count (cells/µL)**		259 [147-404]		257 [118-421]	0.73		236 [117-350]		248 [131-323]	0.94
< 200 cells/µL	188 (34.8)		59 (41.8)		0.12	75 (41.9)		22 (42.3)		0.96
Missing data[1]	/		*1 (0.7)*			/		/		
**Last HIV viral load < 50 copies/mL**	508 (93.9)		129 (90.8)		0.19	170 (95.0)		51 (98.1)		0.46
**Last CD4 count (cells/µL)**		701 [545-900]		708 [492-891]	0.49		712 [539-994]		789 [618-993]	0.27
< 500 cells/µL	111 (21.0)		35 (25.5)		0.25	39 (22.4)		9 (18.0)		0.5
Missing data[1]	*12 (2.2)*		*5 (3.5)*			*5 (2.8)*		*2 (3.8)*		
**Last CD4/CD8 ratio**		0.98 [0.66-1.31]		0.90 [0.62-1.23]	0.17		1.35 [0.83-1.87]		1.08 [0.73-1.59]	0.09
< 1	267 (51.4)		76 (57.1)		0.24	59 (33.9)		20 (40.0)		0.43
Missing data[1]	*22 (4.1)*		*9 (6.3)*			*5 (2.8)*		*2 (3.8)*		
**Third antiretroviral agent**					0.92					0.78
INI	299 (55.3)		76 (53.5)			93 (52.0)		25 (48.1)		
NNRTI	126 (23.3)		35 (24.6)			43 (24.0)		15 (28.8)		
Others	116 (21.4)		31 (21.8)			43 (24.0)		12 (23.1)		
**History of hepatitis C**	86 (15.9)		30 (21.9)		0.13	34 (19.8)		18 (36.0)		0.02
Missing data[1]	*17 (3.1)*		*5 (3.5)*			*7 (3.9)*		*2 (3.8)*		
**Perceived HIV-stigma**					<0.01					<0.01
None	400 (74.2)		70 (50.0)			122 (69.3)		20 (40.0)		
Moderate	96 (17.8)		31 (22.1)			35 (19.9)		13 (26.0)		
Severe	43 (8.0)		39 (27.9)			19 (10.8)		17 (34.0)		
Missing data[1]	*2 (0.4)*		*2 (1.4)*			*3 (1.7)*		*2 (3.8)*		

DD= depressive disorder; INI= integrase inhibitors; IQR=interquartile range; MSM= men who have sex with men; NNRTI= non nucleosidic reverse transcriptase inhibitor

[1] Percentages excluding missing data if present, except for percentages of "Missing data"; [2] p-value of Chi2 or Fisher test for categorical variables and Wilcoxon for continuous variables.

Supplementary Table 4. Socio-demographic, substance use and health characteristics of PLWH (n=903) and general population from EHIS-ESPS survey (n=12,817), in men and women included in "PLWH vs GP analysis".

	Men					Women				
	PLWH (n=675)		GP (n=6,183)		p-value[2]	PLWH (n=228)		GP (n=6,634)		p-value[2]
	N (%)[1]	Median [IQR]	N (%)[1]	Median [IQR]		N (%)[1]	Median [IQR]	N (%)[1]	Median [IQR]	
**Current depressive disorder**	**137 (20.3%)**		**299 (4.8%)**		<0.01	**50 (21.9%)**		**585 (8.8%)**		<0.01
**Age (years)**		56 [48-63]		51 [38-64]	<0.01		54 [48-59]		51 [38-63]	<0.01
**Highest level of education**					<0.01					<0.01
Primary	76 (11.4)		1 444 (23.8)			42 (18.6)		1 813 (27.8)		
Secondary	343 (51.4)		2 836 (46.7)			122 (54.0)		2 747 (42.1)		
University	248 (37.2)		1 795 (29.5)			62 (27.4)		1 971 (30.2)		
Missing data[1]	*8 (1.2)*		*108 (1.7)*			*2 (0.9)*		*103 (1.6)*		
**Employment status**					<0.01					<0.01
Employed	338 (51.0)		3 483 (56.4)			115 (51.1)		3 338 (50.4)		
Unemployed	123 (18.6)		772 (12.5)			70 (31.1)		1 511 (22.8)		
Retired	202 (30.5)		1 922 (31.1)			40 (17.8)		1 778 (26.8)		
Missing data[1]	*12 (1.8)*		*6 (0.1)*			*3 (1.3)*		*7 (0.1)*		
**Net monthly household income (€)** [2]					<0.01					<0.01
<900	170 (27.6)		735 (13.6)			80 (41.2)		968 (16.7)		
900-1499	163 (26.5)		1632 (30.2)			69 (35.6)		1824 (31.5)		
1500-2000	137 (22.2)		1440 (26.7)			33 (17.0)		1497 (25.9)		
>2000	146 (23.7)		1590 (29.5)			12 (6.2)		1499 (25.9)		
Missing data[1]	*59 (8.7)*		*786 (12.7)*			*34 (14.9)*		*846 (12.8)*		
**Partnered**	318 (49.9)		4 880 (78.9)		<0.01	87 (39.4)		4 947 (74.6)		<0.01
Missing data[1]	*38 (5.6)*		0 (0.0)			*7 (3.1)*		0 (0.0)		
**Lack of family ties**	130 (19.5)		288 (4.8)		<0.01	33 (15.3)		252 (3.9)		<0.01
Missing data[1]	*10 (1.5)*		*144 (2.3)*			*13 (5.7)*		*133 (2.0)*		
**Alcohol use**					0.08					0.15
None	87 (13.6)		909 (15.3)			56 (27.6)		2 033 (32.1)		
Without misuse	262 (40.9)		2 168 (36.4)			70 (34.5)		2 300 (36.3)		
With misuse	292 (45.6)		2 876 (48.3)			77 (37.9)		2 010 (31.7)		
Missing data[1]	*34 (5.0)*		*230 (3.7)*			*25 (11.0)*		*291 (4.4)*		
**Age-associated comorbidities***					<0.01					<0.01
0	173 (27.0)		4 146 (70.8)			72 (33.0)		4 554 (72.0)		
1	281 (43.8)		1 165 (19.9)			111 (50.9)		1 109 (17.5)		
2	122 (19.0)		390 (6.7)			30 (13.8)		469 (7.4)		
≥3	65 (10.1)		154 (2.6)			5 (2.3)		190 (3.0)		

Missing data[1]	*34 (5.0)*	*328 (5.3)*		*10 (4.4)*	*312 (4.7)*	
**Pain status**			<0.01			<0.01
None	313 (46.6)	3 653 (60.0)		82 (36.6)	3 472 (53.1)	
Moderate	285 (42.5)	2 069 (34.0)		116 (51.8)	2 509 (38.4)	
Severe	73 (10.9)	369 (6.1)		26 (11.6)	555 (8.5)	
Missing data[1]	*4 (0.6)*	*92 (1.5)*		*4 (1.8)*	*98 (1.5)*	

DD= depressive disorder; GP= General Population; IQR= interquartile range; PLWH= persons living with HIV

[1] Percentages excluding missing data if present, except for percentages of "Missing data"; [2] p-value of Chi2 or Fisher test for categorical variables and Wilcoxon for continuous variables.

[2] Net monthly household income per consumption unit CU (adult=1CU; child>16=0.5CU ; child<16= 0.3CU, treshold for chil at 14 years for EHIS-ESPS participants)

** Age-associated comorbidities among: hypertension, diabetes mellitus type 2, chronic obstructive pulmonary disease, ischemic cardiac event, ischemic cerebrovascular disease.*

Supplementary Table 5. Prevalence ratio of depressive disorder (DD) in PLWH (QuAliV-DD study, n=903) compared to general population (EHIS-ESPS survey, n=12,817) in men and women, pooled and by perceived HIV-stigma severity, according to a-priori-defined block-entry Poisson with robust variance regression models.*

		Model 0*	Model 1*	Model 2*	Model 3*	Model 4*	Model 5*	Model 6*	Model 7*
		PR (95%CI)	PR (95%CI)	PR (95%CI)	PR (95%CI)	PR (95%CI)	PR (95%CI)	PR (95%CI)	PR (95%CI)
**Men (ref: GP)**		–							
**PLWH**		**4.20 (3.48-5.05)**	**4.46 (3.67-5.41)**	**3.89 (3.14-4.82)**	**3.35 (2.70-4.17)**	**3.04 (2.42-3.81)**	**3.16 (2.52-3.95)**	**2.63 (2.06-3.37)**	**2.45 (1.93-3.09)**
p-value[1]		*<0.001*	*<0.001*	*<0.001*	*<0.001*	*<0.001*	*<0.001*	*<0.001*	*<0.001*
Perceived HIV-Stigma	*None*	*3.11 (2.44-3.96)*	*3.27 (2.55-4.20)*	*2.96 (2.27-3.85)*	*2.57 (1.96-3.36)*	*2.39 (1.82-3.15)*	*2.46 (1.87-3.22)*	*1.97 (1.47-2.64)*	*2.02 (1.52-2.69)*
	Moderate	*5.62 (4.15-7.60)*	*5.99 (4.41-8.13)*	*4.72 (3.39-6.58)*	*4.11 (2.94-5.74)*	*3.67 (2.61-5.16)*	*3.88 (2.74-5.49)*	*3.33 (2.32-4.78)*	*2.75 (1.98-3.82)*
	Severe	*8.76 (6.54-11.74)*	*9.42 (7.00-12.69)*	*7.80 (5.63-10.81)*	*6.56 (4.74-9.08)*	*5.71 (4.04-8.07)*	*5.90 (4.21-8.27)*	*5.12 (3.62-7.24)*	*3.51 (2.40-5.41)*
	p-value[1]	*<0.001*	*<0.001*	*<0.001*	*<0.001*	*<0.001*	*<0.001*	*<0.001*	*<0.001*
**Women (ref: GP)**		–							
**PLWH**		**2.49 (1.92-3.22)**	**2.47 (1.89-3.22)**	**2.01 (1.52-2.64)**	**1.69 (1.28-2.23)**	**1.62 (1.23-2.14)**	**1.65 (1.25-2.18)**	**1.49 (1.12-1.98)**	**1.46 (1.09-1.95)**
p-value[1]		*<0.001*	*<0.001*	*<0.001*	*<0.001*	*0.04*	*<0.01*	*<0.001*	*<0.001*
Perceived HIV-Stigma	*None*	*1.47 (0.95-2.27)*	*1.46 (0.93-2.27)*	*1.15 (0.74-1.80)*	*1.01 (0.65-1.58)*	*0.99 (0.63-1.54)*	*1.00 (0.64-1.57)*	*0.90 (0.57-1.41)*	*0.99 (0.62-1.58)*
	Moderate	*3.11 (1.95-4.95)*	*3.06 (1.91-4.91)*	*2.73 (1.73-4.31)*	*2.20 (1.39-3.49)*	*2.12 (1.34-3.36)*	*2.21 (1.39-3.51)*	*1.89 (1.18-3.03)*	*1.60 (0.98-2.60)*
	Severe	*5.42 (3.85-7.63)*	*5.43 (3.79-7.77)*	*4.30 (2.99-6.19)*	*3.41 (2.35-4.96)*	*3.17 (2.16-4.65)*	*3.16 (2.17-4.61)*	*3.01 (2.08-4.36)*	*2.39 (1.54-3.72)*
p-value[1]		*<0.001*	*<0.001*	*<0.001*	*<0.001*	*0.06*	*<0.01*	*<0.001*	*<0.001*

DD= depressive disorder; GP= General Population; M/WLWH= men/women living with HIV; PR= Prevalence ratio (Poisson regression with robust variance)

**Model 0*: unadjusted model; Model 1: adjusted for age; Model 2: Model 1 + level of education, employment status, monthly income; Model 3: Model 2 + partnership status; Model 4: Model 3 + family ties; Model 5: Model 4 + alcohol use; Model 6: Model 5 + age-associated comorbidities; Model 7: Model 6 + pain status*

[1] *p-value of Likelihood Ratio Test between null model and model 0 and then between successive nested models*
